# Oral Administration of Cancer Vaccines: Challenges and Future Perspectives

**DOI:** 10.3390/vaccines12010026

**Published:** 2023-12-26

**Authors:** Marta Gambirasi, Amin Safa, Idris Vruzhaj, Aurora Giacomin, Franca Sartor, Giuseppe Toffoli

**Affiliations:** 1 Experimental and Clinical Pharmacology Unit, Centro di Riferimento Oncologico di Aviano (CRO), IRCCS National Cancer Institute, 33081 Aviano, Italy; marta.gambirasi@cro.it (M.G.); idris.vruzhaj@cro.it (I.V.); fsartor@cro.it (F.S.); 2 Department of Life Sciences, University of Trieste, 34127 Trieste, Italy; aurora.giacomin99@gmail.com; 3 Doctoral School in Pharmacological Sciences, University of Padua, 35131 Padova, Italy; 4 Department of Immunology, School of Medicine, Zabol University of Medical Sciences, Zabol 98616-15881, Iran

**Keywords:** cancer vaccines, mucosal immune system, gastrointestinal barrier, oral administration

## Abstract

Cancer vaccines, a burgeoning strategy in cancer treatment, are exploring innovative administration routes to enhance patient and medical staff experiences, as well as immunological outcomes. Among these, oral administration has surfaced as a particularly noteworthy approach, which is attributed to its capacity to ignite both humoral and cellular immune responses at systemic and mucosal tiers, thereby potentially bolstering vaccine efficacy comprehensively and durably. Notwithstanding this, the deployment of vaccines through the oral route in a clinical context is impeded by multifaceted challenges, predominantly stemming from the intricacy of orchestrating effective oral immunogenicity and necessitating strategic navigation through gastrointestinal barriers. Based on the immunogenicity of the gastrointestinal tract, this review critically analyses the challenges and recent advances and provides insights into the future development of oral cancer vaccines.

## 1. Introduction

Oral cancer vaccines are attracting new attention as a potential tumour immunotherapy strategy to elicit or enhance a specific antitumour immune response against cancer [1]. However, oral administration of cancer vaccines is still a hope in current clinical practice [2]. Nevertheless, oral vaccination should be considered as an alternative to a parenteral strategy; it increases patient compliance and reduces the demand for medical personnel facilitating the diffusion of the vaccine in less developed countries [2]. Furthermore, the administration of oral vaccines plays a significant role in activating the immune system, particularly in relation to the prevention of carcinogenesis through the involvement of the intestinal mucosa. This emerging area of research is both novel and captivating [3]. The gastrointestinal tract (GIT) is widely recognised as the primary immunological site within the human body due to its significant population of lymphocytes, accounting for approximately 70% of the total lymphocyte count. Notably, oral immunisation has the capacity to elicit a dual immune response, involving the activation of the immune system at the intestinal mucosa level through the production of immunoglobulin A (IgA), as well as a systemic immune response mediated by immunoglobulin G (IgG) [2].

The foundation of cancer-vaccine development lies in the process of antigen selection and the choice of vaccination platform. The optimal antigen for the construction of a cancer vaccine should exhibit expression exclusively in cancerous cells while being absent in normal cells. Furthermore, this antigen should be universally present throughout all cancer cells and play a vital role in their sustenance. Furthermore, it is imperative that the tumour antigen possesses a high degree of immunogenicity in order to disrupt immune tolerance and induce a robust and targeted immune response against cancer [4].

Tumour antigens can be divided into tumour-associated antigens (TAAs) and tumour-specific antigens (TSAs). TAAs are self antigens that are highly expressed by tumour cells and absent or low in normal tissues. NY-ESO1 and Glypican-1 (GPC1) are examples of TAAs [5,6]. NY-ESO1, also called New York oesophageal squamous cell carcinoma 1, belongs to the cancer testis antigens (CTAs). The structure of NY-ESO1 suggests that it is involved in cell-cycle progression and growth. This is plausible, as it is physiologically expressed in germ and placental cells during embryonic development. Over time, it is maintained in the spermatogonia and primary spermatocytes, while it decreases in the female oogonia. However, high expression of this TAA is found in numerous cancers, including breast cancer, neuroblastoma, bladder cancer, and hepatocellular carcinoma, suggesting that its physiological function may be advantageous for tumour growth [7]. GPC1 is a member of the HS proteoglycans. It consists of a GPI anchor, a C-terminal, an N-terminal domain, and a secretory signal peptide. It is considered a TAA because it has low expression in adult tissues under physiological conditions but can be overexpressed in cancers, such as pancreatic ductal adenocarcinoma (PDAC), where it plays a role in supporting mitogenic stimuli [6]. The initial development of parenteral cancer vaccines focused on TAAs due to their versatility and potential for broad applicability across diverse patient populations [8]. However, due to their self-antigen property, T lymphocytes that bind these antigens are usually removed by central and peripheral tolerance mechanisms. For this reason, TAAs should be sufficiently immunogenic to break immune tolerance and allow the development of a specific immune response [4].

In contrast, TSAs are specifically expressed by tumour cells. These can be antigens expressed by oncoviruses or neoantigens encoded by cancer mutations. The best example of TSA is KRAS due to its high mutation rate in numerous cancers, such as pancreatic ductal adenocarcinoma (PDAC) and colorectal carcinoma (CRC). The KRAS gene belongs to the rat sarcoma viral oncogene (RAS) family and physiologically regulates several signalling pathways, such as the RAF–MEK–ERK and PI3K–AKT–mTOR pathways, which are particularly involved in cell proliferation, differentiation, and migration. KRAS is so frequently mutated in cancer that it is considered the most common oncogenic gene driver. The most important KRAS mutation is the single-base missense mutation, which preferentially affects codon 12 (G12), codon 13 (G13), or codon 61 (Q61). However, there are also many other mutation subtypes. In general, KRAS mutations impair its control, leading to a constitutively active state. This leads not only to the uncontrolled proliferation of cancer cells, which promotes tumour growth and progression but also to the migration of cancer cells through the epithelial-to-mesenchymal transition (EMT) [9]. TSAs have higher immunogenicity than TAAs, and the T-cell response has limited “off-target” damage and is not affected by immune tolerance. In addition, TSAs have a higher affinity for the major histocompatibility complex (MHC), resulting in a more specific immune response [10]. Due to their peculiar immunogenic potential, cancer vaccines based on TSAs have become the first choice in cancer-vaccine development. While neoantigens have been identified as a potentially effective approach for cancer treatment, a considerable number of these anticipated neoantigens exhibit suboptimal immune responses against tumours. In addition, intra- and interindividual differences between patients limit the use of cancer vaccines targeting neoantigens [8].

In summary, the disruption of immune tolerance and, thus, the activation of a specific antitumour immune response depends on the immunogenicity of the antigen [11] and the choice of the antigen on which the cancer vaccine is based on is essential to the success of the therapy. In addition to the antigen, several crucial factors must be taken into account when choosing a vaccine platform. These factors include the duration of vaccine synthesis, which posed a significant challenge to the development of COVID-19 vaccines, the frequency of inoculations, the accessibility to patients, and the method of administration [5].

At present, there are five distinct strategies under consideration for the development of cancer vaccines. The five types of vaccinations discussed in the literature include peptide-based vaccines, bacteria-based vaccines, nucleic acid-based vaccines, virus-based vaccines, and cell-based vaccines [8,12]. Descriptions are presented in Table 1. Although the development of all these five strategies was based on parenteral administration, some types of vaccination, such as peptides and bacteria-derived vaccines [13], highlight great attention to oral administration [14].

Oral vaccines must be designed to successfully enter the gut, penetrate the mucosal barrier, break immune tolerance, and thus elicit an immune response [15]. Once the vaccine reaches the gut, it must penetrate the intestinal epithelium by four pathways to allow interaction with the underlying mucosa-associated lymphoid tissue (MALT): 1. transmembrane transport, 2. receptor-mediated transport, 3. vector-mediated transport, and 4. transport through microfold cells (M). To facilitate vaccine uptake, different oral vaccine delivery strategies have been investigated, including microparticles (MPs), nanoparticles (NPs), and vaccine adjuvants. This review discusses the physiological and immunological gut aspects of the development of oral cancer vaccines and critically analyses the current strategies leading to new hopes for their clinical application [16].

**Table 1:** Type of cancer vaccines.

Type of Vaccine	Strategy	Advantages	Disadvantages	References
Peptide-based vaccines	They are based on specific epitopes expressed on the surfaces of cancer cells	High specificity	Poorly immunogenic, adjuvants required	[16]
		Tolerable toxicity profile		
Bacteria-based vaccines	Bacteria are genetically engineered to express TSA or TAA	Upon oral administration, probiotics embed and colonize within the preexisting microbiome	Toxicity of bacteria toxins and virulence factors	[12,17]
			Short half-life of bacteria proteins	
			Recombinant plasmid delivered by bacteria might mutate	
Nucleic acid-based vaccines (DNA, RNA)	DNA and RNA vaccines introduce TAA or TSA in host cells to produce specific antigen	They can activate specific and multiple epitopes simultaneously. Inexpensive	Further studies are needed to evaluate the safety and efficacy	[18,19]
Virus-based vaccines	Viral vectors deliver specific antigens to the immune system	Stimulation of both innate and adaptive immune response	Repeated administration might be avoided to disadvantage the induction of antiviral immune responses	[8,19]
		Long-lasting immune response		
		No adjuvants are required		
Cell-based vaccines	Cancer cells elicit a targeted immune response through the inclusion of complete tumour antigens inherent to these cells.	Cell-based vaccines just include the epitopes of both T helper and CTLs	Low immunogenicity	[8,20]
		No selection of a specific tumour antigen is required	The preparation method must maintain the integrity and functionality of antigens	

## 2. Immunological Basis for Oral Vaccination

### 2.1. Fisiology and Immunology of the Intestine

The gastrointestinal tract (GIT) plays a crucial role in the breakdown and assimilation of nutrients derived from meals. The process initiates in the duodenum, where pancreatic juice and bile aid in the digestion of proteins, lipids, and carbohydrates. The jejunum and ileum have the main function of absorbing these nutrients, whereas the large intestine, or colon, is specialised in the absorption of water and the excretion of waste [21].

The intestinal wall, consisting of many layers, plays a vital part in this process. The presence of epithelial cells and villi in the mucosa layer greatly enhances the surface area, hence facilitating efficient absorption. The epithelial cells in the intestinal crypts, derived from stem cells, consist of enterocytes responsible for absorbing nutrients, goblet cells that produce mucus, and Paneth cells that release antimicrobial peptides. The submucosa serves as a framework for support, the muscularis aids in movement, and the serosa covers the gut [22,23].

Gaining a comprehensive understanding of the intricate nature of the intestinal wall is crucial in order to further the development of vaccines for oral cancer. These vaccines need to successfully navigate the challenging gastrointestinal environment and effectively penetrate the mucosal barrier in order to be absorbed and interact with the mucosal immune system.

### 2.2. The Intestinal Immune System

The mucosal lining of the intestines, which has an enlarged surface area, serves a dual purpose in the absorption of nutrients and the protection against pathogens, building upon the knowledge of the intestinal anatomy. The immune system in this area is responsible for maintaining a balance between accepting harmless antigens and protecting against harmful microorganisms in order to preserve the health of the mucosal lining and establish a stable environment in the intestines. Imbalances in this equilibrium can result in conditions such as Crohn’s disease, ulcerative colitis, food allergies, and celiac disease [24,25,26].

**Figure 1:**
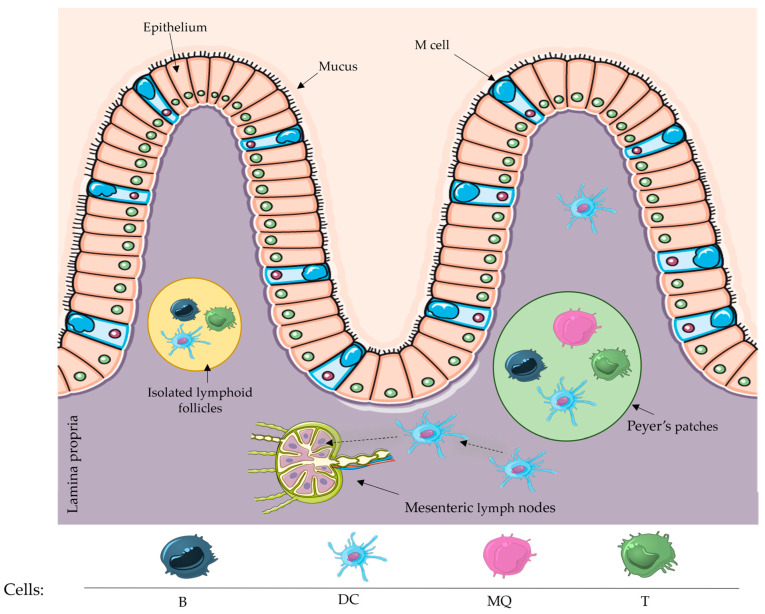
Structure of the intestinal immune system. A single layer of epithelial cells lines the intestine LP. T cells make up the bulk of this epithelial layer, while the lower layer (LP) contains a variety of cell subsets, including B and T cells, macrophages, and cDCs. Interestingly, ILFs and PPs are two specific gut-associated lymphoid structures that contain cDCs. Abbreviations: LP: lamina propria; cDCs: conventional dendritic cells; ILFs: isolated lymphoid follicles; PPs: Peyer’s patches; MQ: macrophages; (This figure was created with smart Sevier Medical art).

The intestinal immune system is divided into two distinct compartments: the inductive compartment and the effector compartment. Inductive sites, such as Peyer’s patches, isolated lymphoid follicles, and mesenteric lymph nodes, serve as the locations where immune responses are launched and where naïve T and B cells are stimulated. The intestinal epithelium and lamina propria play a vital role in carrying out these immune responses [27] (Figure 1).

#### Inductive Sites of the Intestinal Immune System

- Mesenteric lymph nodes

MLNs are situated in the layers of the mesentery. They consist of an outer cortex that contains B and T cells and an inner medulla that contains macrophages and plasma cells. They play a vital role in facilitating the removal of lymph from the intestine, enabling activated lymphocytes to return to the bloodstream and selectively target certain areas inside the intestine, such as the lamina propria (LP) and intraepithelial lymphocytes (IEL). MLNs play a crucial role in starting immune responses in the mucosal surfaces and identifying antigens that are eaten orally. Their contribution to the development of oral tolerance is emphasised by the fact that mice lacking MLNs are unable to establish tolerance to soluble oral antigens [12,28,29];

- Peyer’s patches

Peyer’s patches (PPs) are specialised lymphoid tissues found in the small intestine.

PPs are clusters of lymphoid tissue located in the mucosa of the small intestine, primarily in the ileum. Lymphoid tissues have compartments for B and T cells and are protected by follicle-associated epithelium (FAE), which encompasses microfold cells (M cells) specialised in capturing luminal antigens. The subepithelial dome (SED) located beneath the FAE contains a high concentration of conventional dendritic cells (cDCs). One important characteristic of PPs is the existence of plasma cells that produce IgA, which is essential for the process of IgA class switching. This process is fundamental for maintaining the balance of the intestines by reducing the interaction between bacteria and the mucous membrane that lines the intestines [30,31];

- Isolated lymphoid follicles

Isolated lymphoid follicles (ILFs) are mucosal lymphoid structures that typically comprise a singular aggregation of B cells, lymphoid tissue-inducing cells (LTi), and a small number of T cells, similar to PPs. They come from crypt patches (CPs) and are coated with FAE that contains M cells. The development of ILFs is controlled by the presence of intestinal bacteria, as demonstrated by their impaired development in mice that are free of germs. The potential contribution of dendritic cells to the proliferation and interaction of ILFs through microbial signalling, although important, has not been thoroughly investigated [32,33,34].

### 2.3. Intestinal T Cells

- T cells in the intestinal epithelium

The intestinal epithelium harbours IELs, which are a heterogeneous population of T cells predominantly composed of CD8+ cells. IELs are categorised as either ‘natural’ or ‘induced’ depending on how they are activated and the specific antigens they identify. The amount of induced IELs, consisting mainly of TCRαβ+ CD8αβ+ cells and a smaller population of TCRαβ+ CD4+ T cells, tends to rise as individuals age. These interactions occur in the lymph nodes [35,36,37,38,39]. On the other hand, endogenous IELs, which may be identified by the presence or absence of CD8αα, get activated in the thymus as a result of self-antigen selection. The cells migrate to epithelial tissue at an early stage and remain in a stable population throughout the lifespan [39,40,41];

- T cells in the lamina propria

The LP mostly contains conventional CD4+TCRαβ+ T cells, as well as a few CD8αβ+ TCRαβ+ cells. These T cells, like IELs, frequently display an activated or memory phenotype, which arises from the stimulation of naïve T cells at sites where immune responses are initiated [42]. The colon and gut-associated lymphoid tissue (GALT) are predominantly inhabited by Th17, Th1, and FoxP3+ regulatory T cells. The prevalence of Th2 cells, crucial in defending against parasitic helminths, is reduced in specific pathogen-free mice, possibly as a result of the absence of these parasites. The precise mechanisms and distinct roles of Th1 cells in the intestine, under both normal and inflammatory circumstances, remain incompletely elucidated [42].

### 2.4. Intestinal Dendritic Cells

Dendritic cells (DCs) in the intestine are strategically located across multiple anatomical locations within the lamina propria (LP), encompassing areas such as the PPs and ILFs. Throughout history, there has been considerable debate about the exact definition of cDCs, primarily due to the fact that the markers commonly used to identify DCs are also present in intestinal macrophages. For example, it was discovered that CD11c+MHCII+ cells expressing high levels of CX3CR1, previously classified as DCs, are in fact macrophages arising from monocytes [43,44,45]. True cDCs can be identified by the absence of specific markers, such as FcγR1, CD64, and high levels of CX3CR1 [46]. Within the confines of the small intestine (SI), a significant proportion of CD11c+MHCII+CD64- cDCs exhibit the presence of CD103 (αE), an integrin molecule that interacts with a specific ligand called E-cadherin, which is predominantly localized on the surfaces of small intestinal epithelial cells [45,46]. DCs expressing CD103 can be categorized according to their expression of CD11b [46], where CD103+CD11b- DCs are predominantly found in the lymphoid structures of the intestine, whereas CD103+CD11b+ DCs are mainly localized in the LP. In addition, there is a population of CD103- cDCs in the intestinal region [43,46]. The use of the Zbtb46-GFP reporter mouse model provides evidence that Zbtb46 is a potential marker for colonic cDCs [47]. Research on cDCs in the human gut is still at an early stage. Nevertheless, previous studies have demonstrated the presence of CD103+ DCs in the human small intestine (SI) and MLNs [48,49].

### 2.5. Intestinal Monocytes and Macrophages

Intestinal macrophages, the primary mononuclear phagocytes in the intestine, show significant expression of CX3CR1 and are usually located in close proximity to the epithelial layer within the LP [50,51]. These cells contribute to the maintenance of intestinal homeostasis by removing foreign substances and show a restrained proinflammatory response when stimulated by Toll-like receptors [45,52]. It is worth mentioning that they produce IL-10 and thus facilitate the proliferation of FoxP3+ T cells [45]. These macrophages differentiate from Ly6C^hi^ monocytes and undergo several stages characterized by specific molecular expressions [44,53]. In the context of inflammatory conditions, a specific subset of monocytes is able to perform proinflammatory functions and thus play a role in promoting inflammation [45,54,55].

### 2.6. Microfold (M) Cell Role

The M cells located in the intestinal epithelium play a central role in mediating immune responses by facilitating antigen uptake [56]. M cells are considered one of the most important immune-surveillance factors at the intestinal level due to their involvement in cell uptake in PPs [57]. M cells differentiate from Lgr5+ stem cells in the crypts thanks to the role of RANKL (TNFSF11) expressed by stromal cells in the FAE of GALT [57]. Lgr5+ stem cells are columnar cells at the crypt base of the intestine that are known for their high expression of Wnt target genes and their crucial role in intestinal homeostasis and can differentiate into various intestinal cell types [58,59]. Among these types, M cells differentiate from Lgr5+ stem cells in the crypts, a process facilitated by the role of RANKL (TNFSF11) expressed by stromal cells in the FAE of GALT [57]. M cells are distinguished from other intestinal epithelial cells by their unique morphology. Namely, they are characterized by short and irregular microvilli, known as microfolds, and the invaginated plasma membrane, which forms the structure that harbours immunocompetent cells [56]. In addition, compared to neighbouring epithelial cells, M cells are covered by a thin mucus layer and have small cytoplasmic vesicles, a reduced glycocalyx, and few lysosomes [60]. In addition, several M-cell-specific markers have been identified, including glycoprotein-2 (GP2), the transcription factor Spi-B (Spib), and the chemokine (C-Cmotif) ligand 9 (CCL9) [57]. Due to their properties, M cells are crucial for mucosal immune surveillance, as they transport antigens from the intestinal lumen and represent a gateway to the robust epithelial barrier. M-cell-dependent antigen uptake is usually mediated by specific receptors, such as β1-integrin, cellular prion protein, GP2, and others [61]. In addition, transcriptome analysis of M cells has shown that they express numerous intracellular molecules that may be involved in vesicular transport during the transcytosis pathway. However, how the intracellular transport machinery controls the transcytosis of antigens is unclear and remains to be investigated in detail [56].

**Figure 2:**
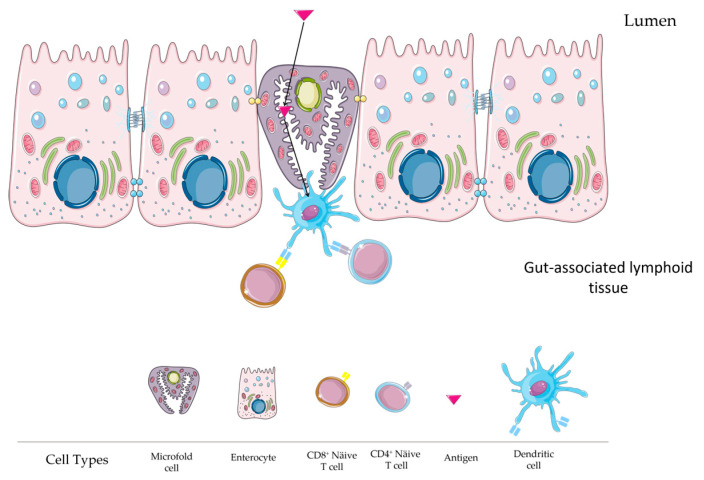
Antigen uptake by M cells and dendritic cells. (This figure was created with smart Sevier Medical art).

Once the antigens are taken up by the M cells, they are transported to the antigen-presenting cells, in particular the DCs. DCs process the antigens, and the fragments are presented on their surfaces for immune activation (Figure 2). Therefore, targeting M cells is a good strategy to improve antigen uptake and thus vaccine efficacy in eliciting a specific immune response [62]. Given the central role of M cells in antigen uptake, understanding and exploiting their function could revolutionize mucosal vaccine delivery.

### 2.7. Cellular and Molecular Mechanisms in Mucosal Immunity Induction via Oral Vaccines

After oral vaccination, a complex series of cellular activities is initiated in both the organised lymphoid tissues and the mucosal lamina propria of the gut. DCs, in conjunction with naïve T and B lymphocytes, coordinate a response to generate antigen-specific antibodies and CTLs, which are essential for protecting mucosal surfaces against infections. sIgA plays a crucial function in protecting the lining of the stomach from harmful microorganisms and counteracting the poisons they release.

#### 2.7.1. The Synthesis and Impact of IgA and IgG in Intestinal Immune Defence

The complex process of IgA antibody synthesis in the gut is coordinated by the collaborative actions of mucosal DCs, T-helper lymphocytes, and B lymphocytes. At first, antigen-loaded DCs in PPs and MLNs release a distinct set of cytokines and stimulatory chemicals, such as TGFβ, IL-10, IL-6, and retinoic acid (a derivative of vitamin A). These molecules have a crucial role in the development of antigen-specific Th2 lymphocytes and Foxp3+ T regulatory cells (Tregs), as described by Coombes [63] and Sun [64]. Tregs can undergo differentiation into T-follicular helper cells (Tfh), as demonstrated by Tsuji [65]. The T-helper cell subsets mentioned in the study [66] play a role in activating B lymphocytes to undergo IgA class switching. This process is facilitated by signals from the B-cell receptor, interactions between CD40 and CD40L, and the presence of certain cytokines, such as IL-4, IL-5, IL-6 IL-10, TGF-β, and IL-21.

In addition, specific DC subtypes in PPs, such as tumour necrosis factor (TNF)/inducible nitric oxide synthase (iNOS)-producing DCs (Tip-DCs), enhance the process of IgA class switching in a manner that does not require T cell involvement. This is achieved by increasing the availability of nitric oxide, which in turn promotes the expression of TGF-β receptors on naïve follicular B cells. These findings were reported in studies [67,68]. Furthermore, it has been observed that Tip-DCs and other subsets of DCs in PPs and MLNs, such as plasmacytoid and follicular DCs, have the ability to stimulate the production of IgA antibodies by B cells in the follicles. This process is not dependent on the interaction between B cell CD40 and CD40L but rather is facilitated by cytokines such as BAFF and APRIL [69]. Furthermore, ILFs and the lamina propria play a crucial role in the initiation of the IgA immune response. Within immune-cell clusters known as ILFs, DCs secrete biologically active TGF-β1. In addition, when stimulated by microbial TLR signalling, DCs, along with other stromal cells, produce BAFF and APRIL [70]. Within the lamina propria, there is a specific subset of DCs that express TLR5 and secrete retinoic acid and IL-6 when exposed to bacterial flagellin. This reaction is accompanied by the secretion of BAFF and APRIL by DCs that express TNF-α and iNOS as a result of TLR signalling [71]. Epithelial cells also have a role in the release of BAFF and APRIL, which promotes T-cell-independent IgA class switching. This was demonstrated by Fagarasan [33] Nevertheless, the functionality of this process is still a subject of discussion in human systems, as highlighted by Berkowska [72].

IgA-committed B cells, generated by T-cell-dependent and -independent pathways, ultimately differentiate into dimeric IgA-producing plasma cells in the lamina propria. This process is controlled by Th2 cytokines IL-5 and IL-6. Following their formation, dimeric IgA antibodies are carried across epithelial cells into mucosal secretions by polymeric Ig-receptor-mediated transcytosis. This process results in the release of sIgA into the gut lumen [73].

SIGA plays a vital role in the immune system by acting as a critical barrier that prevents pathogens from attaching to mucosal surfaces [74]. It attaches to the surface proteins of enteric pathogens, which stops them from colonising and invading. It also plays a role in “immune exclusion” by trapping and removing pathogens in mucus [75]. In addition, IgA has the ability to neutralise bacterial enterotoxins, capture organisms in vesicular compartments within epithelial cells for export and neutralise viruses within cells. This is an important defence mechanism against enteric viruses [76]. Although IgA is the main form of antibody found in mucosal secretions, IgG also has a notable impact on the adaptive immunological defences of the gut. Mostly, IgG enters the gut luminal secretions by passing through from the bloodstream, with a minor amount being produced in the gut itself [74]. Significantly, IgG has the ability to be actively carried through the intestinal epithelium. The transportation process is made easier by a special receptor called FcRn, which is found in intestinal epithelial cells. This receptor facilitates the flow of IgG in both directions across the epithelium [77]. Although FcRn expression declines after weaning in rodents, it continues to be present in absorptive intestinal epithelial cells of humans, pigs, and cattle throughout adulthood [77]. Interestingly, FcRn also carries complexes of IgG and antigens from the inner cavity to the connective tissue, delivering them to cells that present antigens, as proven by Yoshida [78].

Mucosal secretory immunoglobulin M (sIgM), although less abundant compared to secretory sIgA, is also present in stomach secretions. Moreover, IgE, produced by plasma cells in the lamina propria, has a function in defending against particular intestinal helminths by stimulating nearby mast cells [74].

#### 2.7.2. The Role of Cytotoxic T Lymphocytes in Gut Immunity

Cytotoxic T lymphocytes in the mucosa play a central role in the subsequent immune response triggered by oral vaccination [79]. Cytotoxic T lymphocytes play a crucial role in the immune response against invasive pathogens, such as viruses and intracellular bacteria, that enter the body through the mucosal surface. These CTLs are largely produced in PPs by CD8+ lymphocytes, which are stimulated by CD8α+ DCs and/or activated Th1 cells. In mucosal tissues, there exist several populations of CD8+ lymphocytes, with a major presence of those expressing T-cell receptors αβ (TCRαβ) in combination with CD8αβ or CD8αα. These particular populations are mostly associated with effector/cytotoxic activity [80]. CD8αβ+ TCRαβ+ cytotoxic T lymphocytes, as demonstrated by Muller et al. [81], have robust cytotoxic activity against cells infected with viral pathogens and intracellular protozoan parasites. In addition, CD8+ T cells release IFN-γ and TNF-α, enhancing inflammatory responses in the lamina propria. This in turn supports phagocytic cells in destroying bacteria that have broken through the mucosal barrier. For this reason, when developing oral cancer vaccines, it is imperative to define their effect on the CTL immune defence.

#### 2.7.3. Molecular Mechanisms of Gut Homing in Mucosally Primed Effector B and T Cells

Effector B and T lymphocytes activated at intestinal mucosal induction sites have the special ability to migrate back to the mucosal effector sites in the intestine [58,82]. After priming, these cells detach from the stromal cells within the structured lymphoid tissue. They then migrate through the lymphatic system to the MLN, where they undergo further differentiation. They then spread throughout the intestinal mucosa via the bloodstream [59]. The mechanism by which lymphocytes are directed into the intestine is determined by the interactions between the homing receptors on the lymphocytes and the ligands on the vascular endothelium of the intestinal mucosa. Intestinal DCs facilitate the upregulation of intestinal homing receptors, including α4β7 integrin and CCR9 and CCR10 chemokine receptors, on these activated lymphocytes through the secretion of retinoic acid. The receptors in question exhibit binding affinity for various chemicals, such as MadCam-1, CCL25, and CCL28 throughout the GIT [58,82].

On the other hand, lymphocytes activated in peripheral lymphoid organs have specific molecules, such as α4β1-integrin, L-selectin, and CCR4, that hinder their movement towards mucosal surfaces [83]. The elucidation of this unique molecular expression pattern provides an explanation for the ability of oral vaccines, as opposed to injectable vaccines, to induce mucosal immunity.

## 3. Stimulating Intestinal Immune Responses through Oral Vaccination

Following oral vaccination, a cascade of complicated cellular processes is set in motion, which mainly take place in the structured lymphatic tissues and the mucosal LP of the gastrointestinal tract. A number of important immune cells are involved in this particular process, including DCs, naïve T cells, and B lymphocytes [84,85].

These cells work together to generate a targeted immune response against the particular antigen introduced by the vaccine. The main goal of this immune response is the production of antibodies and cytotoxic T lymphocytes (CTLs) specific to the antigen [86]. The above components play a crucial role in establishing mucosal immunity, which is of paramount importance in protecting the GIT from a variety of infections and their harmful byproducts. One of the antibodies produced, secretory immunoglobulin A (sIgA), plays a crucial role in protecting the mucosal surfaces of the GIT [87]. The mechanism of action is that the substance binds to pathogens and their toxins, inhibiting their ability to adhere to and penetrate the gastrointestinal epithelium and thus attenuating their harmful effects [88]. The role of secretory immunoglobulin A (sIgA) is crucial for maintaining the integrity and well-being of the gastrointestinal mucosa [88]. It serves as a basic defence mechanism to protect against gastrointestinal infections and diseases [89]. This immune response is critical to the efficacy and safety of oral cancer vaccines, and great emphasis is placed on the fact that mucosal immunisation elicits a diverse immune response that is separate and distinct from the body’s systemic immune system. The efficacy of an oral cancer vaccine depends on its ability to induce the production of immunoglobulin A (IgA), a critical component of the immune system that helps neutralise infections and malignant cells. In addition to the formation of antibodies, the vaccination must also activate the cell-mediated immune response. This process involves the activation and recruitment of immune cells, such as T cells and natural killer (NK) cells, which have the ability to directly attack and eliminate malignant cells [90].

This response is crucial to combat cells that have undergone malignant transformation and to ensure their efficient identification and elimination. Induction of a systemic antibody response leading to IgG production is in conjunction with local mucosal immunity; the presence of a systemic immune response is of paramount importance. IgG is the predominant antibody isotype found in the circulatory system and serves as a critical component of systemic immunity [91]. This process aids in the recognition and subsequent neutralization of infections and abnormal cells within the whole organism. To ensure complete protection, a successful oral cancer vaccine must necessarily trigger the development of IgG antibodies, thereby extending immunity beyond the gastrointestinal tract [91]. To elicit an adequate immune response, oral vaccines must achieve three main goals: 1. they must successfully enter the intestine without being degraded; 2. they must be transported through the intestinal mucosal barrier; and 3. they must be processed by antigen-presenting cells. To meet these requirements, oral vaccines must overcome the biochemical and physiological challenges of the gastrointestinal microenvironment, which is characterized by the harsh pH gradient, enzymatic degradation, mucosal, and epithelial barriers, and the gastrointestinal microbiota [92].

Although oral vaccines can induce various types of immunogenicity that may lead to effective cancer therapy, the main criticism of oral cancer vaccines is their lower degree of immunogenicity than parenteral vaccines [93]. This could lead to the need to administer a higher dose of antigen compared to parenteral immunization, an aspect that increases the risk of tolerance [18]. Strategies have been developed to improve the immunogenicity of vaccines, including the use of adjuvants, or the modification of amino acid sequences to improve TAA to increase their affinity to HLA molecules [94].

## 4. Commercially Available Oral Vaccines

Oral vaccines provide several advantages, such as increased patient comfort, decreased reliance on medical personnel, and reduced expenses for manufacture and distribution. Self administration is facilitated, removing the requirement for specialised equipment such as needles. This improves patient compliance and decreases healthcare expenses, which can contribute to up to 25% of the total cost of introducing a new vaccination [15,95]. In addition, oral vaccinations are more cost-effective to manufacture compared to injectable vaccines, as they do not necessitate the complex purifying processes required for injection formulations [15]. Their long-term manageability minimises the necessity for frequent hospital visits, therefore mitigating patient anxiety and enhancing their availability, particularly in underdeveloped nations.

Presently, the market comprises oral prophylactic vaccinations designed to combat viral illnesses [96]. Details are reported in Table 2. In 1961, the United States licenced the first oral vaccination for polio, which was created by Sabin. This vaccine has been found to be efficient in generating mucosal antibodies in the intestines of both adults and children [97]. Since 2006, the oral rotavirus vaccine has been accessible in both the United States and worldwide, leading to a notable reduction in rotavirus-related deaths [98]. Oral vaccines for bacterial infections encompass the typhoid vaccine targeting Salmonella enterica, which was released in 2014 and has a 50% protection rate after three years [98]. Additionally, there are many kinds of oral cholera vaccines, including both live attenuated and dead bacteria variants [99]. Although there have been significant improvements, oral cancer vaccines are not now accessible for purchase. However, there is an increasing amount of research being conducted to explore new prospects in this field.

## 5. Oral Administration of Cancer Vaccines

At present, several strategies have been adopted in the development of oral cancer vaccines in mouse models that are summarized in Table 3. Peptides and bacteria-derived oral cancer vaccines have attracted the greatest interest since peptides can be easily protected and delivered to the gut by various strategies [103], Furthermore, the inherent existence of microorganisms inside the gut microbiome offers a distinct prospect for the advancement of vaccines [104].

Peptides can be used in vaccine cancers by mimicking known or predicted tumour antigen epitopes, which may consist of short or long peptides. Short peptides are directly presented by MHC-I molecules without APC processing. However, they are not considered the best choice for cancer vaccines as they have a short half-life in vivo and are restricted by HLA types. In contrast, long peptides have to be processed by APCs. Therefore, after internalization, parts of long peptides are degraded via the endosomal pathway, while other parts enter the cytoplasmic or vacuolar pathway. The first pathway leads to the loading of fragments onto MHC-II molecules and thus to the activation of CD4+ T cells, while the second pathway leads to cross presentation by MHC-I molecules and thus to the priming of CD8+ T cells [5]. The advantage of long peptides is related to their multiple epitopes that allow broader coverage of HLA. Moreover, long peptides are able to elicit a more specific antitumour immune response than short peptides. Finally, conversely to short peptides, which are of chemical-synthesis derivation [8], long peptides are usually produced by protein-expression systems such as Escherichia coli, plants, yeasts, and insect and mammalian cells [8].

Table 3 provides references to several studies that have shown the potential of oral peptide-based cancer vaccines in inducing targeted immune responses against tumours. However, other studies have found that peptide vaccines have limited immunogenicity [86,105,106,107]. This problem may depend on the limitation of the MHC polymorphism and the design of the peptides. For this reason, the use of immunostimulatory adjuvants and delivery systems is essential [108]. Muhammad Khairul Amin et al. have shown that PLGA nanoparticles functionalized with sodium alginate (ALG) or eudragitol (EUD) can protect peptides from gastric pH and reach immune cells in the intestine [21].

Nowadays, bacteria are considered a promising platform for an oral cancer vaccine, since bacteria constitute the gut microbiota, which has a pivotal role in immunogenicity against cancer [109]. Bacteria can be genetically modified to express and deliver antigens, genes, therapeutic proteins, or molecules, thus activating both innate and adaptive immune responses [12,15]. The antigens produced by the engineered bacteria have a wide range of effects on the immune system by interacting with Toll-like receptors (TLRs), promoting the secretion of inflammatory cytokines and chemokines, upregulating co-stimulatory molecules, activating specific T helper and cytotoxic T cells, and suppressing of regulatory T cells in the tumour microenvironment. In addition, the use of bacteria has many advantages in terms of easy and inexpensive large-scale manufacturing. Presently, pathogenic species (e.g., *Salmonella Typhimurium*; *Escherichia coli; Listeria*; *Clostridium*; *Corynebacterium*; *Pseudomonas*); and nonpathogenic species (e.g., *Lactobacillus*; *Lactococcus*; *Bifidobacterium*) are among the bacteria being explored for cancer immunotherapy [15]. Among these, the efficacy of Bifidobacterium longum has been observed in various types of cancer, such as renal carcinoma, bladder cancer, and glioblastoma, as outlined in Table 4. *Listeria monocytogenes* and lactic acid bacteria have demonstrated potential in the field of cervical cancer and carcinoembryonic antigen overexpressing cancer therapy, respectively [110,111]. The involvement of *E. coli* in the development of colorectal cancer and the ability of Salmonella typhimurium to effectively target various cancer antigens, such as NY-ESO-1 and ovalbumin, emphasize the potential of oral vaccine administration as a strategy to treat a wide range of cancers [112,113].

Investigating nucleic acid-based vaccines: the utilisation of DNA vaccines in the field of cancer treatment is currently undergoing a significant transformation. The development of these vaccines, which have been meticulously engineered to specifically target essential cancer antigens, is revolutionising the field of cancer therapy by opening up novel avenues for treatment. An example of a unique approach in addressing cancer-related angiogenesis involves the utilisation of bacterial vectors coated with NPs for the delivery of DNA vaccines targeting VEGFR2 [87]. Similarly, the strategic oral administration of Salmonella enterica in the context of lung cancer therapies specifically targeting VEGFR-3 serves as a notable example of the inventive implementation of nucleic acid-based vaccines [114]. The utilisation of adeno-associated viral (AAV) vectors represents a notable progression in the field of virus-based cancer therapy. In the context of breast cancer, the vaccines under consideration mostly target the neu antigen. These vaccines represent a promising development, highlighting the efficacy of virus-based approaches in initiating robust antitumour responses [115].

Advances in cell-based vaccines: cell-based vaccines have become an important component in cancer immunotherapy. The vaccines, which are characterised by their special composition, demonstrate their importance in the fight against many forms of cancer. The use of whole-cell lysates and enteric polymers in the context of ovarian cancer has opened up new possibilities for therapeutic interventions [116]. The use of oral microparticulate vaccines to improve the immunotherapy of prostate cancer is an example of the flexible and responsive properties of cell-based methods [117].

Strategies for oral vaccines based on the administration of therapeutic nucleic acids have been also considered, particularly in diseases of the gastrointestinal tract, including colon and rectal cancer. However, the development of oral nucleic acid vaccines is hampered by their instability, low cellular uptake, and immunogenicity [118,119]. These problems are due to the high molecular weight of nucleic acids, their negative charge, and their hydrophilicity [119]. For this reason, research on oral nucleic acid-based vaccines should also focus on efficient delivery systems that protect the vaccine and on adjuvants that facilitate the breakthrough of immune tolerance [120]. Table 3 lists examples of studies of oral DNA-based cancer vaccines for melanoma, breast cancer, and lung cancer. In these studies, the vaccine is not administered alone but is protected by bacteria or NPs to improve efficacy and then tested in mice.

The first study on viral vectors for oral cancer vaccines was by Jason C. Steel et al. This group of researchers used AVV vectors for the oral delivery of vaccines against the neu oncogene in a breast cancer model. They focussed on AVVs due to their ability to be unaffected by pH and temperature, low toxicity, and natural tropism for DCs. In the study, mice treated with a single oral dose of two different AVV-neu vectors developed an immune system against breast cancer cells and improved their survival [115]. Finally, cell-based oral cancer vaccines have also been reported in the literature. The same challenges of stability and immunogenicity need to be overcome. For this reason, this type of vaccine also relies on delivery systems, as shown in Table 3.

**Table 3:** Comprehensive overview of oral cancer vaccines: types, targets, and therapeutic outcomes.

Type of Oral Cancer Vaccine	Subtypes	Cancer	Antigen	Delivery System	Species	Outcome	Ref
Peptide-based vaccines	Long peptide	Colorectal cancer	AH1	Liposome	Mouse	Oral emulsion vaccine reduces colorectal tumour	[103]
	-	OVA-expressing tumours	Ovalbumin	ovalbumin as the antigen encapsulated in whole glucan particles	Mouse	WGP-OVA: A Promising Cancer Vaccine	[121]
	-	B cell lymphoma A20	MHC class-I restricted peptides derived from survivin	Oral co-administration of β-glucan with peptide vaccination	Mouse	β-Glucan Enhances Peptide Vaccination Efficacy	[122]
Bacteria-based vaccines	Bifidobacterium longum	Renal Carcinoma	WT1	Oral Bifidobacterium	Mouse	Enhances RCC Immunotherapy	[104,110]
	Bifidobacterium longum	Bladder cancer	WT1	Oral Bifidobacterium	Mouse	Enhances Bladder Cancer Immunotherapy	[123]
	Bifidobacterium longum	Glioblastoma	WT1	Oral Bifidobacterium	Mouse	Shows Promise for Glioblastoma Treatment	[124]
	Bifidobacterium longum	Prostate cancer	WT1	Oral Bifidobacterium	Mouse	Promising Oral Cancer Vaccine (B440) for Prostate Cancer Treatment	[125]
	Bifidobacterium longum	Wilms’ Tumour 1	WT1	Oral Bifidobacterium	Mouse	Induces Effective WT1-Specific Immunity	[125]
	Bifidobacterium longum	Leukaemia	WT1	Oral Bifidobacterium	Mouse	Superior Antitumour Effect of WT1 Oral Cancer Vaccine Over Peptide Vaccine	[126]
	Bifidobacterium longum	Acute myeloid leukemia	WT1	Oral Bifidobacterium	Mouse	Shows Strong Antitumour Activity	[127]
	Listeria monocytogenes	Cervical cancer	HPV-16 E7	Oral Listeria	Mouse	Cervical Cancer Treatment	[110]
	Lactic acid bacteria	Cancers overexpressing carcinoembryonic antigen	CEA	Oral LAB	Mouse	LABs as Oral Vaccine Vector for CEA Antigen	[111]
	*E. coli*	Colorectal Cancer		Tumour antigen- and adjuvant-containing emulsions or liposomes	Mouse	Oral Bacteria Biohybrid-Based Vaccines for Colorectal Cancer Treatment	[112]
	Salmonella typhimurium	Pancreatic cancer	VEGFR2	Oral Salmonella	Human	Monthly Boost Vaccinations with VXM01 in Advanced Pancreatic Cancer Patients	[128]
	Salmonella typhimurium	targets cancers expressing the NY-ESO-1 tumour antigen	NY-ESO-1	Oral Salmonella	Mouse	Salmonella Typhimurium-Based Vaccine for Cancer Immunotherapy	[113]
	Salmonella typhimurium	Cancer expressing ovalbumin (OVA)	OVA	Oral Salmonella	Mouse	H_2_O_2_-Inactivated Salmonella Typhimurium RE88 as a Novel Cancer Vaccine Carrier	[95]
	Salmonella typhimurium	Hepatocellular carcinoma (HCC)	AFP	Oral Salmonella	Mouse	Therapeutic Efficacy of an Oral DNA Vaccine Against Hepatocellular Carcinoma	[129]
Nucleic acid-based vaccines	DNA vaccine	cancer-related angiogenesis	VEGFR2	nanoparticle-coated bacterial vectors	Mouse	Nanoparticle-Coated Bacterial Vectors for DNA Vaccines	[87]
	DNA vaccine	Lung	VEGFR-3	Oral administration using attenuated Salmonella enterica serovar typhimurium strain SL3261	Mouse	Oral VEGFR-3-Based Vaccine for Lung Cancer Inhibition	[114]
	DNA vaccine	Melanoma	Heat shock protein 70 (Hsp70) and tumour-associated antigens (TAA)	Oral administration using attenuated Salmonella typhimurium strain SL3261 as the carrier	Mouse	Oral Hsp70-TAA DNA Vaccine for Cancer Immunotherapy	[130]
	DNA vaccine	Breast	Murine endoglin (CD105)	Oral delivery using double attenuated Salmonella typhimurium (dam −, AroA −)	Mouse	Oral Endoglin DNA Vaccine for Breast Cancer	[131]
	DNA vaccine	Melanoma	Plasmid DNA gp100	Oral delivery using a nanogel (Alg-Tat-gp100)	Mouse	Multi-Faceted Nanogel for Oral DNA Vaccine	[132]
Virus-based vaccines	Adeno-Associated Viral Vector (AVV)	Breast cancer	*neu*	AAV	Mouse	Effective Antitumour Vaccination	[115,123]
Cell-based vaccines		Melanoma Colon cancer		Oral particulate	Mouse	Potential for Effective Antitumour Responses	[133]
		Ovarian	Whole-cell lysate	enteric polymers	Mouse	Effective Against Ovarian Cancer	[116]
		Prostate cancer	Whole-cell lysate	Aleuria aurantia lectin	Mouse	Effective Oral Vaccine Against Prostate Cancer	[134]
		Prostate	TRAMP-C2	Oral microparticulate vaccine	Mouse	Oral Microparticulate Vaccine Enhances Prostate Cancer Immunotherapy	[117]
		Breast		microparticles combined with intraperitoneal administration of cyclophosphamide	Mouse	Effective Breast Cancer Immunotherapy	[135]

## 6. Challenges in Oral Delivery: Gastrointestinal Barriers

Cancer vaccines must reach the small intestine in an active form, but above all in the correct fold, in order to increase the likelihood that they will trigger a specific immune response. However, proteins and peptides can be inactivated or degraded on their way through the GIT due to a number of chemical and physiological barriers. These include a harsh pH environment, enzymatic degradation, mucus and epithelial barriers, and the gastrointestinal microbiota [92].

In addition, immunogenicity can be impaired by nonexhaustive antigen selection, tolerance mechanisms, and an immunosuppressive tumour microenvironment (TME) at the tumour site [136].

### 6.1. Harsh pH Environment

**Figure 3:**
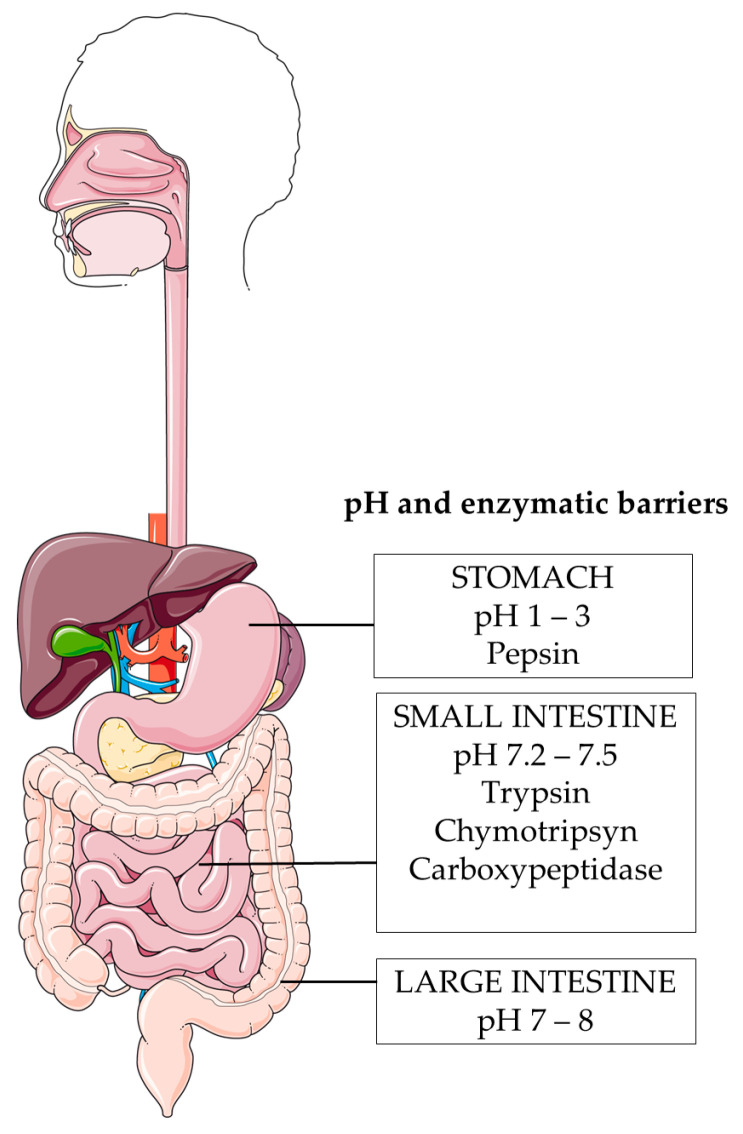
pH and enzymatic barriers for antigens. (This figure was created with smart Sevier Medical art).

The first limit for the oral administration of vaccines is the pH gradient that characterises the GIT. Each section of the GIT is characterised by a different pH, ranging from 1 to 8. Specifically, the pH is 1–3 in the stomach, around 7 in the small intestine, and between 7 and 8 in the large intestine [137,138] (Figure 3).

This range can change over time and depends on food intake and pathological conditions. The pH of the stomach is higher after birth, then decreases rapidly and remains constant throughout life [139]. In addition, the pH of the stomach increases after food intake and the pH of the colon varies from person to person depending on the individual diet [140]. In addition, pathological conditions, particularly colon cancer, inflammatory bowel disease (IBD), ulcerative colitis (UC) [141], and Chron’s disease (CD) [142] influence the pH value in the colon.

The stability of DNA, RNA, and proteins is influenced by the pH gradient. While DNA is stable in a broader pH range, such as the physiological range, RNA is unstable at an alkaline pH and stable at a pH of 4–5, and proteins and peptides are stable in a pH range close to their pI value but can be unfolded and oxidised, hydrolyzed, or deamidated, losing their activity [143]. In general, bacteria can be affected by gastric pH, but they can survive by activating mechanisms to respond to stress conditions [144].

As a result, cancer vaccines are sensitive to different sites of the GI system, depending on the pH. It is imperative to protect the vaccine to prevent its inactivation and degradation.

### 6.2. Enzymatic Degradation

The stability of oral cancer vaccines in the GIT involves the enzymatic barrier (Figure 3). Enzymes, both endogenous and those synthesized by the host bacteria, are essential for the catalyzed digestive reaction [145]. The main enzymes that can modify antigens in the GI tract are secreted by the stomach, pancreas, and small intestine. However, it is important to consider other enzymes such as luminal enzymes, mucosal enzymes, and enzymes secreted by bacteria in the large intestine [140].

The most important enzymes that act in the stomach are pepsins. They are peptidases that work under very acidic conditions and catalyze cleavage at specific sites in proteins and peptides [146], leading to the hydrolysis of peptide bonds as well as nucleic acids and bacterial membranes [140].

Pancreatic enzymes include trypsin, chymotrypsin, carboxypeptidase A, carboxypeptidases B and elastase, which lead to the rapid degradation of peptides [140]. For example, insulin has been shown to be degraded by pancreatic enzymes within one hour of administration [146].

When the peptides or nucleic acids reach the small intestine, they are processed by other enzymes such as aminopeptidases, endopeptidases, and enteropeptidases. Finally, the products are ready to be absorbed and enter the blood capillaries [147].

This change may affect the potency and efficacy of peptide-based cancer vaccines, so the enzymatic barrier must be taken into account in the development of vaccines.

### 6.3. Mucus Barrier

The GIT is entirely lined by mucus, which plays a vital role in maintaining body homeostasis. The main constituents of it are water, lipids, electrolytes, proteins, and other elements [148]. Mucins, the primary structural proteins, are extensively glycosylated glycoproteins that greatly contribute to their viscoelasticity [149,150]. There are two categories of mucins: transmembrane mucins, which play a role in the development of glycocalyx, and gel-forming mucins, which are produced by goblet cells [151,152]. MUC2 is the primary mucin responsible for the formation of gel-like substances [150]. Mucus functions as a protective barrier against bacteria, poisons, enzymes, and antigens. The size of antigens directly impacts their capacity to permeate this barrier [153]. MUC2 has the ability to attach to DCs in the small intestine, thereby affecting the process of presenting antigens and promoting immunological tolerance [154]. To overcome the obstacle of delivering peptide-based cancer vaccines, researchers have been investigating the use of mucoadhesive materials such as polymethacrylate-based copolymers and chitosan [155,156]. These materials aim to enhance the delivery of the vaccine by improving its ability to penetrate the barrier.

### 6.4. Epithelial Barrier

The epithelial barrier, a cell layer lining organs and body cavities, acts as a crucial shield against pathogens and harmful substances..

The intestinal epithelium, consisting of many cell types, such as enterocytes, Paneth cells, goblet cells, and M cells, serves as the primary physical barrier against foreign antigens. Molecules traverse this epithelium through transcellular, carrier-mediated, or paracellular routes. Tight connections between epithelial cells [157,158] govern the permeability of the paracellular route. Efficient operation of these channels enables the assimilation of nutrients while preventing the entry of foreign substances [36]. To effectively stimulate an immune response, oral cancer vaccines should be specifically formulated to target enterocytes, especially M cells [2,85].

### 6.5. Gastrointestinal Microbiota

The gut microbiota, consisting of more than 1000 bacterial species, exhibits substantial variation among individuals, exerting an impact on drug metabolism, toxicity, and effectiveness [159]. These changes can impact therapeutic proteins and peptides by influencing drug metabolism, deactivating medicines, and altering the expression of metabolic enzymes [160,161]. The microbiota can also influence the development of genetically modified bacteria employed in oral bacterial vaccines [125,127].

## 7. Strategies to Overcome the Challenges of Oral Administration of Cancer Vaccines

The search for, investigation, and application of strategies to overcome gastrointestinal barriers that hinder the efficient oral administration of cancer vaccines is essential for the clinical translation of them.

Despite the many challenges to this goal, several areas can be explored in depth to find solutions that will make a cancer vaccine an excellent candidate for oral delivery. To overcome gastrointestinal barriers, the first strategy to consider is an appropriate delivery system that protects the vaccine from the pH and enzymes in the gastrointestinal tract. Then, one must consider the need to promote the crossing of the vaccine through the mucosal and epithelial barriers. This aspect can be addressed by investigating strategies to target enterocytes, M cells, and APCs. Finally, the use of adjuvants can also help in activating the immune response, especially in the case of weakly immunogenic cancer vaccines, such as those based on TAAs.

### 7.1. Delivery Systems

Different types of delivery systems have been proposed to protect oral vaccines in the gastrointestinal tract and overcome its barriers in terms of pH and enzymes, but also to improve mucus penetration and, therefore, the uptake by cells deputed to activation of immune response. Delivery systems can be divided into live and nonlive vaccines [162].

Living delivery systems include recombinant bacteria and viral vectors. Due to their PAMP, they are recognized by the PRRs of the host cells and trigger an immune response. They therefore act as effective adjuvants. Among recombinant bacteria, lactic acid bacteria and Salmonella strains are the most promising [163], while adenoviruses are the most promising viral vectors [164].

Nonliving delivery systems include virus-like particles (VLPs), MPs, and nanoparticles. These vaccine platforms are less immunogenic but have a greater capacity to protect the vaccine during passage through the GIT [162]. Specifically, coated VLPs—molecules that resemble the virus but do not contain genetic material [165] have been shown to protect the vaccine from the harsh gastrointestinal environment, preserve the properties of the vaccine, and promote the breakdown of immune tolerance [166].

Micro- and nanoparticles, especially those synthesized from polymers, such as chitosan [167] and poly(lactic-co-glycolic acid) (PLGA), protect the internalized material from degradation in the gastrointestinal tract. Polymeric materials enhance the internalisation of particles by APCs and thus contribute to the stimulation of the immune system [122,168].

The use of Aleuria aurantia lectin (AAL), derived from the edible orange peel fungus A. aurantia, is gaining recognition as an innovative method for vaccine delivery [169]. Due to its strong attraction to α-l-fucose and its structural similarity to neuraminidases found in other infections, this particular entity has a remarkable ability to selectively target M cells. Consequently, this ability leads to enhanced immune responses when tested in mice [170]. In parallel, there is growing interest in the use of liposomes, which are tiny lipid bilayer structures, as effective carriers for antigens in the field of vaccine production [119]. The remarkable aspect of their functionality lies in their ability to protect antigens, accommodate a wide range of antigen types, and enhance the immune response by precise delivery to the antigen-presenting cells [171].

In addition, glucans derived from various sources, such as plants, microbes, and synthetic materials, have been shown to be very effective as adjuvants in the delivery of vaccines [172]. The remarkable ability of these substances to elicit a broad spectrum of immunological responses, including the formation of antibodies without adverse consequences, is of great importance [173]. In particular, the use of NPs enables effective loading of antigens and precise binding to antigen-presenting cells.

In conclusion, the choice of an appropriate delivery system is essential to ensure proper vaccine functionality.

### 7.2. Targeting Strategies

In addition to adjuvants, strategies for overcoming the mucosal and epithelial barrier by oral vaccines regard the targeting of enterocytes, M cells, and antigen-presenting cells. These cells are characterized by various markers that can be used to promote the uptake of oral vaccines and trigger an immune response in the endogenous GALT. Some M-cell markers in particular are clusterin, cathepsin E [174], glycoprotein 2 (GP2) [175], sialyl Lewis A antigen [176], and complement 5a receptor (C5aR) [177]. In terms of targeting enterocytes, the most important marker investigated is the neonatal Fc receptor (FcRn). It is able to mediate the internalisation of IgG antibodies through transictosis by interacting with their Fc segment. The validity of this idea was demonstrated by Pridgen, E. M. and Co, who targeted the intestinal epithelium with FcRn-targeted nanoparticles. They demonstrated that targeted NPs achieved an absorption efficiency of 13.7% compared to 1.2% for nontargeted NPs [178]. Finally, APCs, especially dendritic cells in the gut, can be targeted to stimulate their maturation and activation. Oral vaccines can be delivered to DCs by adding PAMPs or other receptor ligands such as lectins or FcRn to their formulation [85,116].

#### Directing Attention towards DC Surface Receptors:

DEC205 and Clec9A are essential receptors found on the surfaces of dendritic cells (DCs), which are responsible for delivering cancer vaccines [179,180]. DEC205 plays a crucial role in immunological responses, namely in CpG responses [181]. The lack of it in mice leads to hindered development of cDC1 cells, decreased production of cytokines, and weakened CD8+ T-cell responses [181]. The impact is associated with the crucial role of the intracellular domain of the receptor in the process of receptor endocytosis [182,183,184]. Ligands that are attached to DEC205 are transported to late endosomes/lysosomes, resulting in the efficient presentation of antigens on MHC class I and II molecules [185]. The significance of DEC205 in vaccine-delivery techniques is underscored by this key mechanism. The selective activation of CD4+ and CD8+ T cells can be achieved by targeting the model antigen OVA to DEC205, particularly when combined with adjuvants such as anti-CD40 mAb, CpG, polyIC, or LPS [186,187]. This approach has demonstrated efficacy in safeguarding against mouse models of melanoma and breast cancer [188,189].

Clec9A plays a unique and supplementary role in antigen presentation [190,191,192]. It selectively directs antigens to early and recycling endosomes, which helps in presenting dead-cell-associated antigens to T cells through cross-presentation [191,192,193]. Clec9A functions as an endocytic receptor in DCs, unlike DEC205, although it does not activate them [190,191]. The ligand of this molecule is F-actin, which is present on necrotic cells and recognised as a damage-associated molecular pattern (DAMP) [194,195]. Targeting Clec9A, especially when paired with adjuvants, has been demonstrated to stimulate strong CD8+ T-cell responses, which are equally effective as those triggered by DEC205 targeting [191,196]. Furthermore, Clec9A plays a crucial role in stimulating CD4+ T-cell responses, which are vital for facilitating CD8+ T-cell responses in cancer immunotherapies. Studies have shown that, by directing antigens like OVA or MHCII-restricted OVA epitopes towards Clec9A, coupled with adjuvants, it effectively triggers both CD4+ and CD8+ T-cell responses [190,197]. This highlights its substantial capability in eliminating tumours in mice models.

By strategically targeting the receptors DEC205 and Clec9A, there is a great opportunity to improve the effectiveness of cancer vaccines when administered orally. This presents a new potential in cancer immunotherapy.

### 7.3. Adjuvants

Adjuvants are substances that stimulate the immune response to improve the efficacy of the vaccine; they can therefore be used to overcome the low immunogenicity of oral cancer vaccines. Several oral adjuvants have been investigated in recent years, including toxin derivatives, pathogen recognition receptor (PRR) ligands, pathogen-associated molecular patterns (PAMPs), gut peptide cell adjuvants, and cytokines [198]. A summary is reported in Table 4.

The best-studied toxin derivatives are the cholera toxin (CT) and heat-labile enterotoxin (LT), as they are able to activate antigen-presenting cells and promote an IgA response [97]. These toxins could enhance antigen permeation through the intestinal epithelial barrier [162] and promote the differentiation of intestinal stem cells into M cells, improving mucosal immune surveillance thanks to their specific role [199].

However, CT and LT have a high toxicological profile in humans. Therefore, multiple mutant CT (mmCT) and double mutant LT (dmLT), modified versions of CT and LT, have been developed to maintain efficacy as adjuvants but reduce toxicity [101,102].

PAMPs are recognized by the corresponding PRRs, which play a critical role in host defence against pathogens. PRRs can be divided into Toll-like receptors (TLRs) [200], NOD-like receptors (NLRs) [201], and RIG-I-like receptors (RLR) [202,203], but C-type lectin receptors (CLRs) are also involved in microbial recognition [203]. The cell types that express them include intestinal epithelial cells and antigen-presenting cells. Several researchers have shown that PAMPs in oral vaccines can enhance the immunogenicity of the vaccine and increase the production of IgA and IgG antibodies [5].

**Table 4:** Adjuvants for oral vaccines.

Type of Adjuvants	Subtypes	References
Toxin derivatives	Cholera toxin (CT)	[204]
	labile enterotoxin (LT)	[205]
Pattern-Recognition Receptor (PRR) ligands	Toll-like receptor (TLR) ligands	[206]
	NOD-like receptor (NLR) ligands	[207]
	RIG-I-like receptor (RLR) ligands	[208]
	C-type lectin receptor (CLR) ligands	[209]
Gut Peptide Cell Adjuvants	M-Cell Peptides/Ligands	[210,211,212]
Cytokine Adjuvants	IL-1β, IL-2, IL-12	[213,214,215]

## 8. Conclusions

The oral administration of cancer vaccines is becoming a revolutionary approach in the field of cancer therapies. For years, the focus has been on vaccines developed for parenteral injection. However, the transition to oral administration is gaining momentum, mainly due to its unparalleled advantages. Oral administration simplifies the process, is cost-effective, and expands the possibilities for global distribution, making treatment more accessible to diverse populations.

However, the journey of an oral vaccine is not without its challenges. On their journey through the GIT, these vaccines face a number of obstacles ranging from fluctuating pH levels to a variety of enzymes and the ever-changing gut microbiota. These obstacles jeopardise the integrity and efficacy of the vaccine.

Innovative solutions are being developed to overcome these challenges. The introduction of adjuvants aims to improve the immunogenicity of the vaccine. In addition, research is being conducted to develop vaccine platforms that specifically target enterocytes, M cells, and antigen-presenting cells to ensure effective delivery and uptake. In addition, the use of protective delivery mechanisms, such as microparticles and nanoparticles, provides an additional layer of security to protect the vaccine as it passes through the gastrointestinal tract. To effectively combat the immunosuppressive environment of the tumour, it may be necessary to combine vaccine therapies with other treatments as the simultaneous administration of vaccines together with immune-checkpoint inhibitors or other immunomodulatory agents. This combination therapy aims to counteract the mechanisms by which the tumour evades the immune system [95]. The integration of multiomics approaches is crucial for advancing the development of oral cancer vaccines. It allows for a full investigation of genomes, proteomics, and metabolomics. This technique is crucial for the identification of precise biomarkers, comprehension of tumour microenvironments, and customisation of individualised therapeutics.

Oral cancer vaccines have the potential to redefine the landscape of cancer treatment. In addition to their therapeutic efficacy, they promise to improve patient adherence and significantly reduce healthcare costs. This burgeoning field invites increased research efforts and promises a brighter, more hopeful horizon for cancer patients around the world.

## Figures and Tables

**Table 2:** Commercially available oral vaccines.

Disease	Vaccine Type	Manufacturer	Trade Name	References
Poliomyelitis	Poliovirus vaccine inactivated	Sanofi Pasteur, Paris, France	IPOL^®^	[26,29]
Rotavirus	Live attenuated monovalent human rotavirus strain	GlaxoSmithKline	Rotarix^®^	[100]
	Pentavalent live vaccine (lyophilized)	Serum Institute (India)	Rotasiil^®^	[101]
	Pentavalent live vaccine	Merck and Co., Inc.	RotaTeq^®^	[102]
Cholera	Cholera toxin B subunit and inactivated V. cholerae 01 whole cells	Valneva	Dukoral^®^	[102]
	Live attenuated V. cholerae 01 strain (CVD 103.HgR)	PaxVax	Vaxchora^®^	[102]
Typhoid	Ty21a live attenuated vaccine	Previously PaxVax Berna GmbH, now Emergent Biosolutions	Vivotif^®^	[98]
